# Caffeine before cesarean delivery: a novel preventive strategy against spinal hypotension, a double blind placebo-controlled trial

**DOI:** 10.1186/s44158-025-00333-z

**Published:** 2026-01-15

**Authors:** Mina Adolf Helmy, Kerlous Adolf Helmy, Rana M. Zaki, Sara A. Khatab, Sherif Alaa Embaby, Reham Amin Kaddah, Mohamed Ahmed Shamma, Lydia Magdy Milad

**Affiliations:** 1 Department of Anesthesia and Critical Care Medicine, Cairo University, Cairo, Egypt; 2 Department of Obstetrics, Gynecology and Perinatology, Sechenov University, Moscow, Russia; 3 Lecturer of Anesthesia and Critical Care Medicine, Faculty of Medicine, Cairo University, Cairo, Egypt

**Keywords:** Caffeine, Spinal anesthesia-induced hypotension, Ephedrine, Cesarean delivery, Maternal hemodynamics

## Abstract

**Background:**

Spinal anesthesia-induced hypotension is a common complication during cesarean delivery, often requiring vasopressor support, and is associated with maternal discomfort. Caffeine, a central nervous system stimulant with well-documented cardiovascular effects, may provide a simple adjunct to enhance hemodynamic stability. We aimed to evaluate the efficacy of a single preoperative 200 mg oral caffeine dose in reducing the incidence and severity of hypotension following spinal anesthesia in healthy patients undergoing elective cesarean delivery.

**Methods:**

In this randomized controlled trial, 90 patients classified as ASA II and scheduled for elective cesarean delivery under spinal anesthesia were assigned to receive either 200 mg oral caffeine or a placebo 30 min before the procedure. Hemodynamic parameters, the incidence and severity of hypotension, baseline and 60 min post-administration serum caffeine levels, ephedrine requirements, incidence of postoperative nausea and vomiting, and post-dural puncture headache were recorded and analyzed.

**Results:**

Caffeine administration significantly reduced the incidence of hypotension (9% vs. 33%, *p* < 0.05). Severe hypotension was not observed in the caffeine group. Patients in the caffeine group demonstrated greater hemodynamic stability, with a delayed onset of hypotension and reduced ephedrine requirements. No significant differences were observed in the incidence of bradycardia, tachycardia, or reactive hypertension. Neonatal outcomes were comparable between the groups. Additionally, caffeine was associated with lower rates of postoperative nausea and vomiting (2% vs. 20%) and post-dural puncture headache (2% vs. 16%) at 24 h.

**Conclusion:**

Preoperative administration of 200 mg oral caffeine is a cost-effective strategy for reducing spinal anesthesia-induced hypotension, the incidence of postoperative nausea and vomiting, and post-dural puncture headache in healthy patients undergoing elective cesarean delivery. These findings support further investigation of the role of caffeine as an adjunct in obstetric anesthesia.

**Trial registration:**

The study was registered by the principal investigator (M. Helmy) at ClinicalTrials.gov under the identifier NCT07076654 on July 11, 2025.

**Graphical Abstract:**

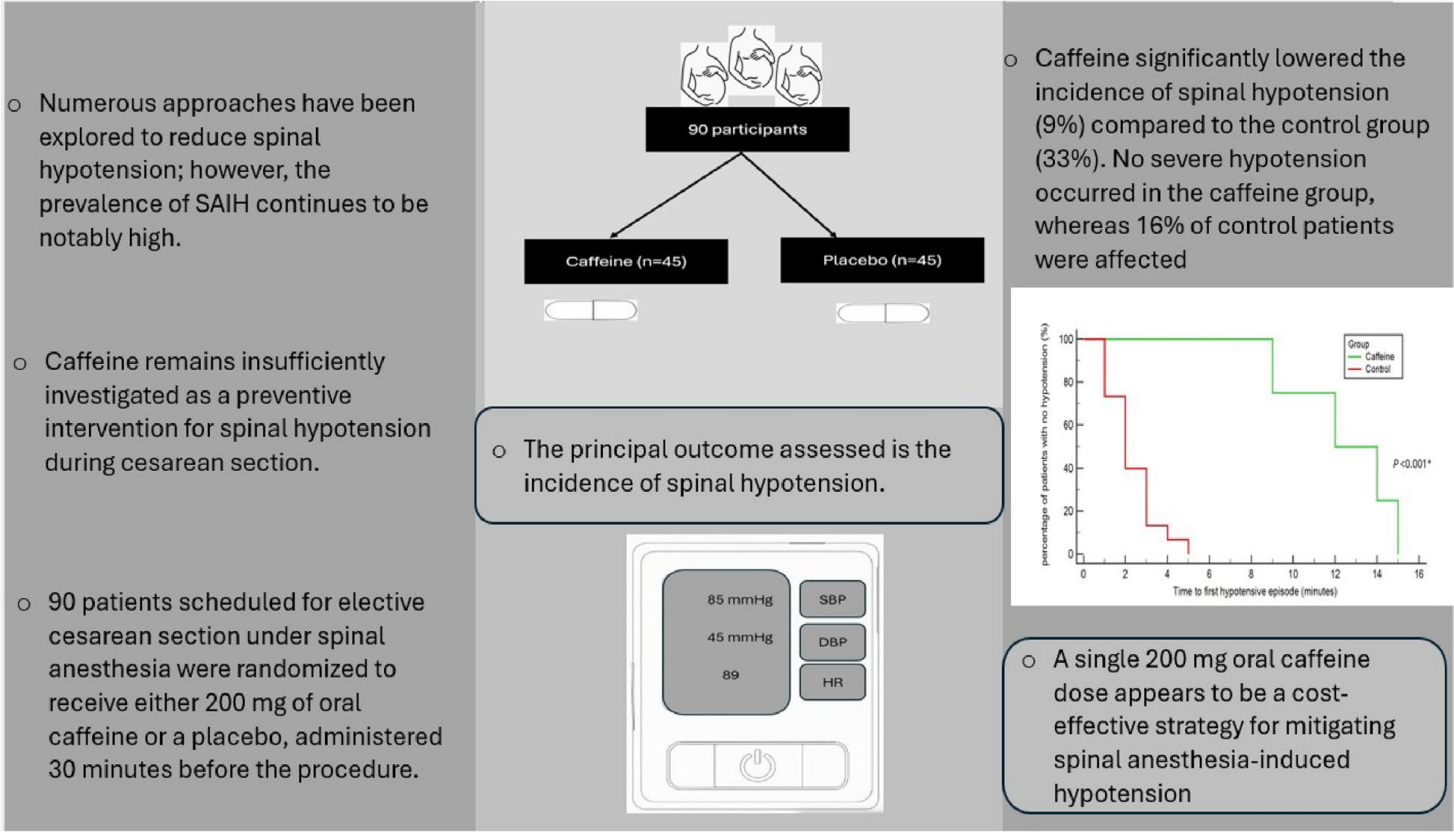

## Highlights

- This is the first randomized trial to assess the influence of preoperative 200 mg oral caffeine on the incidence and severity of spinal anesthesia-induced hypotension.
- 200 mg of oral caffeine reduces the incidence and severity of spinal-induced hypotension.
- Beyond its hemodynamic effect, it reduces the incidence of postoperative nausea and vomiting and post-dural puncture headache.

## Introduction

Spinal anesthesia is the preferred regional technique for obstetric procedures due to its rapid onset, high maternal satisfaction, and avoidance of airway manipulation [1, 2]. However, spinal anesthesia-induced hypotension (SAIH) remains a common and significant complication, with an incidence reaching up to 70%. SAIH is defined as a reduction in systolic blood pressure (SBP) exceeding 20% from baseline or an absolute SBP below 100 mmHg [2–4].

In obstetric settings, SAIH may lead to adverse maternal outcomes, including hypoperfusion-related neurological and renal impairment. Furthermore, compromised uteroplacental perfusion can result in neonatal acidosis and bradycardia. These risks highlight the importance of early prediction and prompt management of SAIH [2]. Various strategies have been proposed to mitigate SAIH, including fluid preloading and co-loading, vasopressor administration, and mechanical interventions such as left uterine displacement and leg elevation [2, 3, 5]. Despite these measures, the incidence of SAIH remains substantial, indicating a continued need for simple, safe, and non-invasive prophylactic approaches.

Caffeine, a naturally occurring methylxanthine, is widely consumed as a dietary supplement. It is readily available, cost-effective, and generally associated with a low incidence of adverse effects. Caffeine exerts mild cardiovascular stimulatory effects by increasing circulating catecholamine levels and antagonizing adenosine receptors (A1, A2A, and A2B), thereby promoting vasoconstriction [6]. Following a single oral dose of 80 to 300 mg, studies have demonstrated increases in SBP by approximately 3–8 mmHg and diastolic arterial pressure by 4–6 mmHg. These hemodynamic effects typically commence within 30 min of ingestion, peak between 60 and 90 min, and return to baseline within 2 to 4 h [6–9].

Despite its widespread use and well-established safety profile, caffeine has not been extensively studied as a prophylactic agent against spinal hypotension during cesarean delivery. Therefore, this study aimed to evaluate the efficacy of a single 200 mg oral dose of caffeine, administered 30 min before spinal anesthesia, in preventing hypotension in healthy pregnant women undergoing elective cesarean section.

## Patients and methods

This randomized controlled trial was conducted at a university hospital following approval by the Institutional Review Board of Cairo University Hospitals (N‑175‑2025) on July 10, 2025, and was registered by the principal investigator (M. Helmy) at ClinicalTrials.gov under the identifier NCT07076654 on July 11, 2025. This study was conducted in accordance with the CONSORT guidelines. Randomization was performed using computer-generated numbers concealed in opaque and sealed envelopes. These envelopes were opened by a research assistant who was not involved in the study. Written informed consent was obtained from all participants prior to their enrollment. The inclusion criteria consisted of pregnant women classified as ASA physical status II, aged 20–40 years, with a singleton pregnancy, and scheduled to undergo elective cesarean delivery under spinal anesthesia. Patients were eligible if they met these criteria and had no contraindications to spinal anesthesia. Exclusion criteria included hypertensive disorders of pregnancy, such as chronic hypertension, gestational hypertension, and preeclampsia, as well as refusal to participate, a baseline systolic blood pressure below 100 mmHg, a known allergy to caffeine, or any contraindication to spinal anesthesia. In addition, cases with an inadequate spinal block height, defined as a sensory level below the T4 dermatome, were excluded from the analysis. Fasting status was confirmed as 6 h for solids and 2 h for clear fluids. To minimize confounding effects, the patients were instructed to abstain from caffeine intake for at least 12 h before surgery. All patients were premedicated with an intravenous injection of pantoprazole (40 mg) and ondansetron (4 mg). Demographic data, such as age, gestational age, weight, and body mass index (BMI), were recorded for all participants.

### Patient allocation

Participants were randomly assigned to one of two groups. The caffeine group (*n* = 45) received a 200 mg caffeine capsule (Caffeine 200 mg, Organic Nation, Cairo, Egypt) with sips of clear water 30 min before spinal anesthesia. The control group (*n* = 45) received a placebo capsule, identical in size, shape, and color, prepared by the same manufacturer, 30 min before spinal anesthesia.

Upon arrival in the operating room, an 18-gauge intravenous cannula was inserted into the forearm vein. Standard monitoring was performed, including electrocardiography, pulse oximetry, and noninvasive blood pressure measurement. A blood pressure cuff was placed on the arm opposite the IV access. Blood pressure was recorded at 1-min intervals until delivery and every 3 min thereafter. Baseline SBP was calculated as the average of three consecutive measurements with less than 10% variation, taken with the patient in a supine position and a 15-degree left lateral tilt. Thresholds of 60%, 80%, and 120% of the baseline SBP were calculated for outcome assessment.

### Anesthesia protocol

Spinal anesthesia was administered in the sitting position at the L4–L5 interspace using a 25-gauge Quincke-type spinal needle (Kindly Medical Devices Co., Ltd., China). The interspace was identified using surface anatomical landmarks, specifically Tuffier’s line, which corresponds to the L4 vertebral body or the L4–L5 interspace.

The intrathecal injection included 10 mg hyperbaric bupivacaine (Sunny Pivacaine; Bupivacaine HCl 20 mg/4 mL, Sunny Medical, Cairo, Egypt) and 25 µg fentanyl (Fentanyl-Hameln; 100 µg/2 mL, Hameln Pharmaceuticals, Germany).

Sensory block height was assessed five minutes after injection and before the surgical incision using bilateral cold sensation testing with an alcohol-soaked cotton swab. A target sensory level of T4 was established.

All patients received a co-load of 400 mL lactated Ringer’s solution over 10 min. A continuous infusion of noradrenaline at 0.08 µg/kg/min (Norepinephrine-Mirola, INAD Pharma for Mirola, Cairo, Egypt) was initiated and maintained until delivery. Lactated Ringer’s solution was administered at 10 mL/kg/h throughout the procedure.

Spinal hypotension (SBP < 80% of baseline) was treated with 6 mg boluses of ephedrine. Bradycardia (heart rate < 60 bpm) was treated with atropine 0.5 mg. In cases of reactive hypertension characterized by an SBP exceeding 120% of baseline, the noradrenaline infusion was transiently stopped until blood pressure returned to baseline, after which the infusion was resumed at the same fixed rate.

### Blinding protocol

To ensure double blinding, the capsules were prepared to be identical in size, shape, and color. Group allocation was concealed using opaque, sealed envelopes opened by a research assistant who was not involved in any other aspect of the study. The patients, anesthesiologists administering spinal anesthesia, operating surgeons, and data collectors responsible for recording intraoperative and postoperative outcomes were all blinded to group assignment. This blinding protocol was maintained throughout the perioperative period to minimize bias and ensure the integrity of the study outcomes.

### Data collection and study outcomes

Hemodynamic parameters, including systolic blood pressure and heart rate, were continuously monitored and recorded during the procedure. Neonatal assessments included the APGAR score and umbilical artery pH, both measured five minutes after delivery. Venous blood samples (3 mL) were collected from each participant at two time points: immediately before capsule administration (baseline, C₀) and 60 min post-administration (C₁). Samples were drawn using standard aseptic technique into serum separator tubes and centrifuged at 3000 rpm for 10 min. The resulting serum was stored at − 80 °C until analysis.

Serum caffeine concentrations were determined using high-performance liquid chromatography (HPLC) with ultraviolet detection, following a validated protocol.

The difference between these two measurements (C₁ − C₀) was calculated for each patient to assess the changes in caffeine concentration over time.

The primary outcome of this study was the incidence of hypotension, defined as a decrease in systolic blood pressure to less than 80% of the baseline value. Secondary outcomes included the incidence of severe hypotension (SBP < 60% of baseline), the time to the first hypotensive episode, the occurrence of bradycardia, defined as a heart rate below 60 beats per minute, and occurrence of significant tachycardia defined as an elevation in heart rate exceeding 130% of the individual's established baseline, in the absence of confounding factors such as hypotension or ephedrine administration [10]. The study also evaluated the incidence of reactive hypertension, as well as neonatal outcomes, including the APGAR score and umbilical artery pH at five minutes. Additionally, maternal side effects were assessed, including the incidence of postoperative nausea and/or vomiting (PONV) within the first 24 h postoperatively, and the occurrence of post-dural puncture headache (PDPH) during the same period.

### Sample size

The sample size was determined using G*Power software based on the incidence rate of spinal hypotension, the primary outcome measure in this study. According to a recent study [1], The reported incidence of spinal hypotension is 32.5%. Assuming a 25% reduction in this incidence would be clinically significant and setting the statistical power at 0.80 with a 5% alpha error, the minimum required sample size was calculated to be 86 participants in total (43 per group). To account for potential dropouts, the sample size was increased to 45 patients per group.

### Statistical analysis

Normality of data distribution was assessed using the Shapiro–Wilk test. Accordingly, data are presented as mean ± standard deviation for normally distributed variables or as median with interquartile range (Q1 to Q3) for skewed data. Group comparisons were performed using unpaired t-tests for normally distributed continuous variables and Mann–Whitney U tests for non-normally distributed continuous variables. Categorical variables were analyzed using the chi-square test; however, when expected cell frequencies were less than 5 in 2 × 2 contingency tables, Fisher’s exact test was applied to ensure statistical validity. Serial changes in SBP were evaluated using a two-way ANOVA to examine both time and group effects. The time to the first hypotensive episode was analyzed using Kaplan–Meier survival analysis, and differences between groups were assessed using the log-rank test. Baseline characteristics were summarized as mean ± standard deviation, and median (quartiles). Because this study is a randomized controlled trial, no hypothesis testing was performed for baseline comparisons. Instead, absolute standardized differences (ASDs) were calculated to assess baseline balance between groups. ASDs were computed as the difference in group means divided by the pooled standard deviation. This approach follows recommended methodological guidance for randomized trials [11]. Secondary outcomes were grouped into predefined families to account for multiple comparisons, and Bonferroni correction was applied within each family using the direct adjustment method. Specifically, raw *p*‑values were multiplied by the number of outcomes in the corresponding family. The hemodynamic family included seven outcomes (incidence of hypotension, incidence of severe hypotension, time to first hypotensive episode, ephedrine use, incidence of hypertension, bradycardia, and tachycardia). The postoperative family comprised two outcomes (PONV and PDPH). The neonatal family included five outcomes (PaO₂, PCO₂, base excess, Apgar score, and NICU admission). The primary outcome was analyzed without multiplicity correction, while both unadjusted and adjusted *p*‑values for secondary outcomes are reported side by side and interpreted cautiously as exploratory findings. All statistical analyses were conducted using SPSS Statistics software, version 26.0 (IBM Corp., Armonk, NY), and MedCalc Statistical Software, version 20.218 (MedCalc Software Ltd., Ostend, Belgium), with statistical significance defined as a two-sided *P*-value less than 0.05.

## Results

**Fig. 1:**
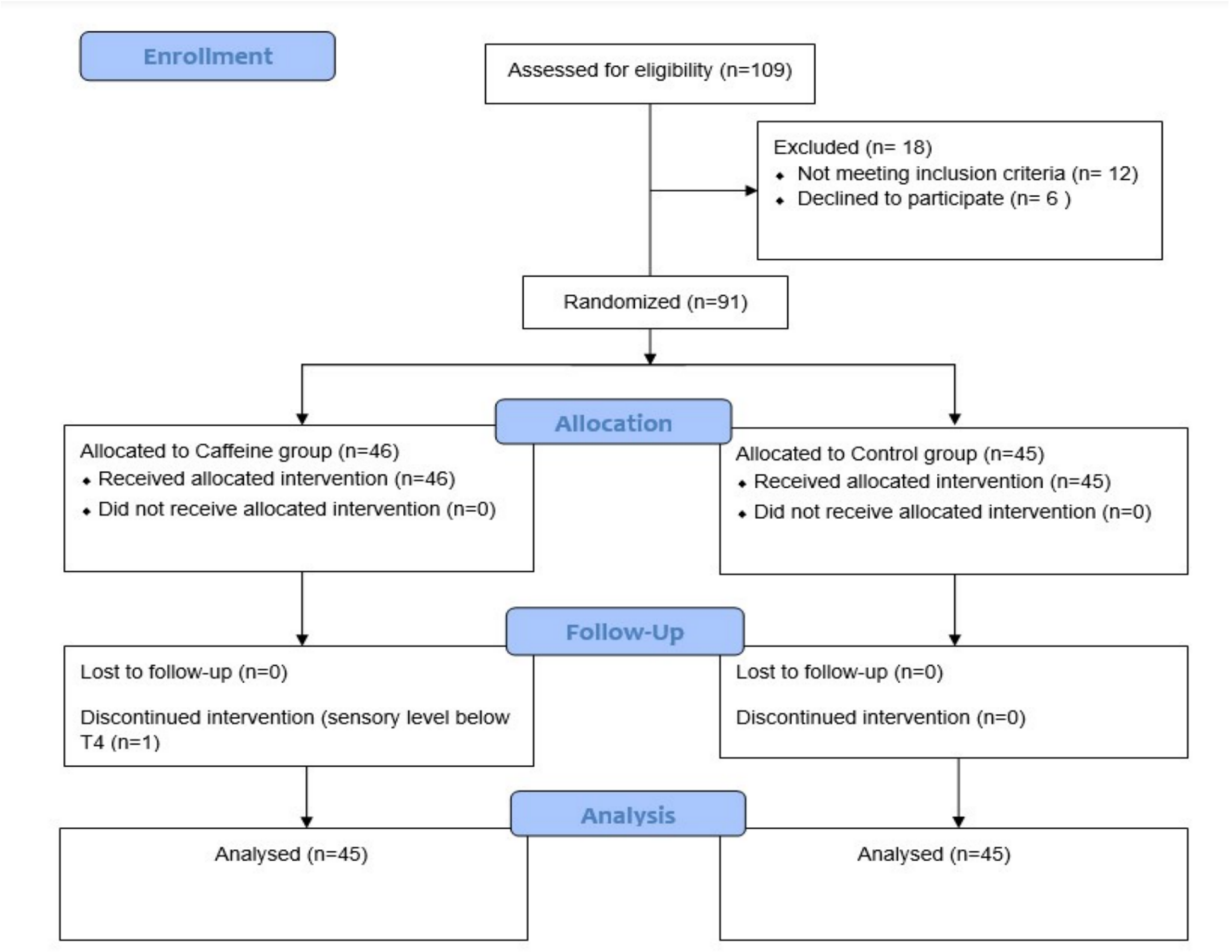
Patient enrollment

A total of 109 patients were screened for eligibility. Of these, 18 were excluded based on predefined criteria. The remaining 91 patients were enrolled and randomized into two groups: 46 patients were assigned to the caffeine group and 45 to the control group. One patient was subsequently withdrawn from the study due to an inadequate sensory level (below T4), as illustrated in Fig. 1. Baseline demographic and clinical characteristics, including age, weight, gestational age, baseline heart rate, systolic blood pressure, estimated blood loss, baseline serum caffeine level, and time to delivery, were summarized in Table 1.

**Table 1:** Demographic data

	Caffeine group (*n* = 45)	Control group (*n* = 45)	Absolute standardized difference
Age (years)	29 $\pm$ 4.9	30 $\pm$ 5.4	0.19
Weight (Kg)	68 $\pm$ 9	69 $\pm$ 9	0.11
Height (m)	1.6 (1.5–1.6)	1.6 (1.5–1.7)	0.00
BMI (Kg/m^2^)	27.2 $\pm$ 2.4	26.7 $\pm$ 2.2	0.22
Gestational age (weeks)	38.5 (37.5–38.5)	38.5 (37.5–39)	0.00
Baseline HR (beats/min)	84 $\pm$ 9	86 $\pm$ 9	0.22
Baseline SBP (mmHg)	124 $\pm$ 5	123 $\pm$ 5	0.20
Baseline serum caffeine level (mg/L)	1.58 $\pm$ 0.81	1.39 $\pm$ 0.71	0.25
Caffeine serum level 60 min post administration (mg/L)	9.43 $\pm 2.27$	0.75 $\pm$ 0.39	5.26
Difference in caffeine level (C1-C0) (mg/L)	7.85 $\pm$ 1.89	−0.64 $\pm 0.49$	6.11
Induction to delivery time (minutes)	16 (14–18)	16 (15–17)	0.00
EBL (ml)	650 (573–730)	680 (575–775)	0.21

Data presented as mean $\pm$ standard deviation, and median (quartiles)

*BMI* Body mass index, *C0* Baseline caffeine serum level, *C1* Caffeine serum level 60 min post-administration, *EBL* Estimated blood loss, *HR* Heart rate, *SBP* Systolic blood pressure

**Fig. 2:**
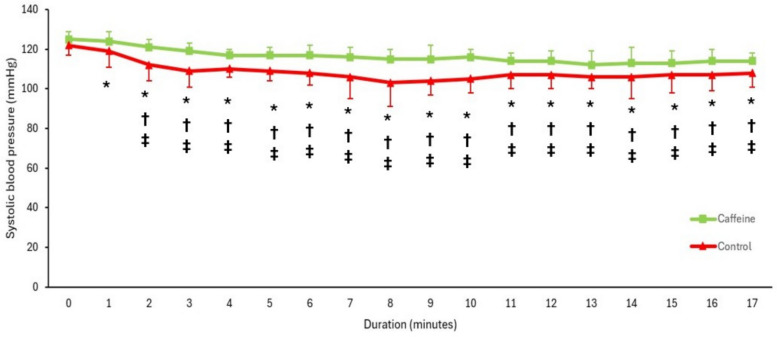
Systolic blood pressure over time. * Denotes a significant difference compared with the other group. † Denotes a significant difference compared with baseline for the caffeine group. ‡ Denotes a significant difference compared with baseline for the control group. Dots represent the mean systolic blood pressure, and error bars indicate the standard deviation

SBP declined progressively over time in both groups; however, the magnitude of reduction was notably greater in the control group (Fig. 2). The incidence of hypotension was significantly lower in the caffeine group (9%) than in the control group (33%), and no cases of severe hypotension were observed in the caffeine group, whereas 16% of patients in the control group experienced severe hypotension (Table 2).

**Table 2:** Maternal outcomes

	Caffeine group (*n *= 45)	Control group (*n* = 45)	Unadjusted *P* value	Adjusted *P* value
Time to first episode (minutes)	13 (10–15)	2 (1–3)	0.002*	0.014*
Incidence of overall hypotension	4/45 (9%)	15/45 (33%)	0.009*	––––
Incidence of severe hypotension	0	7/45 (16%)	0.012*	0.084
Hypotensive episodes				
• One • Two • Three	3/45 (7%) 1/45 (2%) 0	5/45 (11%) 6/45 (13%) 4/45 (9%)	0.021*	0.147
Hypertension	1/45 (2%)	0	> 0.999	> 0.999
Bradycardia	0	3/45 (7%)	0.242	> 0.999
Tachycardia	0	1/45 (2%)	> 0.999	> 0.999
Ephedrine (mg)	6 (6–6)	18 (12–24)	0.003*	0.021*
PONV	1/45 (2%)	9/45 (20%)	0.007*	0.014*
PDPH	1/45 (2%)	7/45 (16%)	0.026*	0.052

Data presented as median (quartiles), and count (percentage)

*PDPH* Post-dural puncture headache, *PONV* Postoperative nausea and vomiting

^*^ Denotes statistical significance

**Fig. 3:**
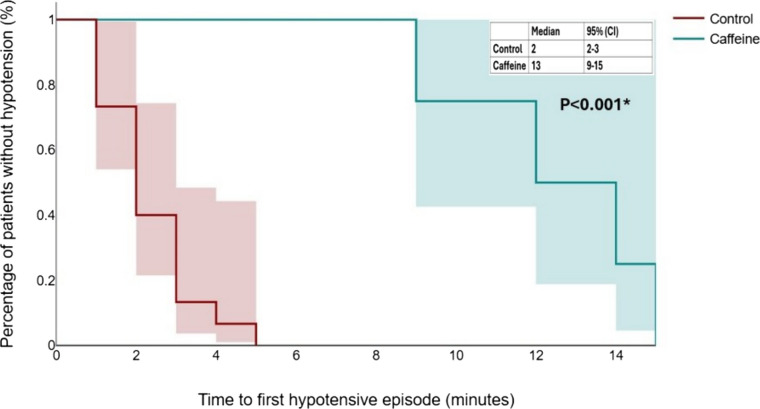
Kaplan–Meier curve depicting time to first hypotensive episode in both study groups, the shaded regions indicate the 95% confidence intervals

The incidence of hypertension, bradycardia, and tachycardia did not differ significantly between the groups (Table 2). Neonatal outcomes were comparable between the two groups (Table 3). Kaplan–Meier analysis revealed a significantly longer time to the first hypotensive episode in the caffeine group (*P* < 0.001; Fig. 3), further supporting the hemodynamic stability associated with caffeine administration.

**Table 3:** Neonatal outcomes

	Caffeine group (*n *= 45)	Control group (*n* = 45)	Unadjusted *P* value	Adjusted* P* value
APGAR score at 5 min	9 (9–10)	9 (8–10)	0.348	> 0.999
Umbilical artery pH	7.29 (7.28–7.31)	7.29 (7.26–7.31)	0.563	> 0.999
Base excess (mmol/L)	−2.5 to + 2	−2 to + 2	0.916	> 0.999
PCO_2_	46(41–50)	46(36–48)	0.213	> 0.999
NICU admission	0/45 (0%)	0/45 (0%)	> 0.999	> 0.999

*NICU* Neonatal intensive care unit

**Fig. 4:**
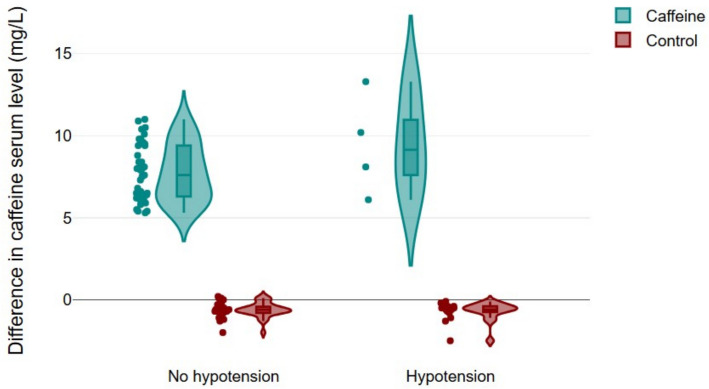
Box-violin plot illustrating the distribution of caffeine serum level differences between patients with and without hypotension in both groups. Each dot represents an individual patient, providing a detailed view of the data

Notably, the Caffeine group demonstrated a markedly lower incidence of PONV and PDPH, with rates of 2% and 2% compared to 20% and 16%, respectively, in the control group. A combined box-and-violin plot was used to compare the differences in caffeine serum levels between patients with and without hypotension (Fig. 4). Each data point represents an individual patient, allowing for the visualization of both the central tendency and distribution.

## Discussion

This randomized controlled trial demonstrated that preoperative administration of a single 200 mg oral caffeine dose significantly reduced both the incidence and severity of spinal anesthesia-induced hypotension in patients undergoing elective cesarean delivery. Compared to the placebo group, patients receiving caffeine exhibited greater hemodynamic stability, with a notably lower incidence of hypotension (9% vs. 33%) and complete absence of severe hypotension (0% vs. 16%). These findings support the potential role of caffeine as a simple, cost-effective adjunct to spinal anesthesia in obstetric patients.

The attenuation of blood pressure decline observed in the caffeine group may be attributed to its pharmacologic actions, namely, adenosine receptor antagonism and increased catecholamine release, which collectively enhance vascular tone and cardiac output [7, 8]. Kaplan–Meier analysis further demonstrated a longer time to the first hypotensive episode, reinforcing caffeine’s stabilizing influence during the perioperative period. Importantly, the incidence of bradycardia, tachycardia, and reactive hypertension did not differ significantly between groups, suggesting that caffeine did not induce excessive cardiovascular stimulation. Neonatal outcomes, including APGAR scores and umbilical artery pH, were comparable, indicating no adverse effects on neonatal well-being.

In line with our findings, recent work [12] has shown that preoperative ingestion of a 200 mg caffeine tablet also reduces the incidence of spinal-induced hypotension, the number of hypotensive episodes per patient, and ephedrine requirements within the first hour of spinal anesthesia for lower limb orthopedic procedures. These results suggest that the hemodynamic benefits of caffeine may extend beyond obstetric populations.

Beyond its cardiovascular effects, caffeine was associated with a markedly lower incidence of PONV (2% vs. 20%) and PDPH (2% vs. 16%) than placebo. These outcomes are consistent with previous reports of caffeine’s efficacy in managing PDPH and suggest additional postoperative advantages that may enhance maternal comfort and recovery [13].

Talih et al. [14] utilized a cup of coffee as the caffeine source, administered two hours before spinal anesthesia. In contrast, our study employed a standardized 200 mg oral caffeine capsule administered 30 min before the procedure. This timing more closely aligns with caffeine’s pharmacokinetic profile, which reaches peak serum concentrations within 30 to 60 min, thereby optimizing its physiological impact. To confirm this, we measured serum caffeine levels at baseline and again 60 min post-administration. Moreover, the use of a fixed-dose capsule ensured uniform caffeine exposure across participants, minimizing variability. The randomized design of our study further enhances its methodological rigor and bolsters the internal validity of our findings. Caffeine was administered in capsule form to ensure precise dosing and eliminate variability associated with coffee consumption. Unlike brewed coffee, which contains a range of bioactive compounds such as chlorogenic acids, diterpenes, and trace minerals that may influence cardiovascular or neurological responses [15], caffeine capsules provide a standardized, pure form of caffeine without additional confounding ingredients.

### Limitations

Although the results are promising, several limitations must be acknowledged. The study was conducted at a single center, and although it was adequately powered for the primary outcome, the sample size may have limited its generalizability. Additionally, the study population consisted exclusively of healthy ASA II patients with singleton pregnancies, and the findings may not apply to patients with comorbidities or high‑risk pregnancies such as gestational hypertension, preeclampsia, or multiple gestations. Moreover, neonatal caffeine concentrations were not measured, despite caffeine’s known ability to cross the placenta; documenting fetal exposure would provide important insights into potential subclinical or long‑term effects. Outcomes were only assessed up to 24 h postoperatively for mothers (PONV, PDPH) and limited to immediate neonatal metrics (umbilical artery pH, Apgar score), leaving uncertainty about the sustained safety profile of caffeine. Future research should investigate optimal dosing strategies, timing of administration, and potential interactions with other anesthetic agents. Larger multicenter trials that include high‑risk populations, incorporate long‑term maternal and neonatal follow‑up, and measure neonatal caffeine concentrations will be essential to validate and extend these findings.

## Conclusion

A single 200 mg oral caffeine dose appears to be a cost-effective strategy for mitigating spinal anesthesia-induced hypotension and improving postoperative outcomes, namely PONV and PDPH, in healthy pregnant women undergoing elective cesarean delivery. Its low cost, ease of administration, and favorable safety profile make it a promising adjunct in obstetric anesthesia, warranting further investigation in broader patient populations.

## Data Availability

The datasets generated and analyzed during the current study are available from the corresponding author upon reasonable request.
